# Diagnostic value and mediation effects of the visceral adiposity index, triglyceride-glucose index, and platelet-to-HDL ratio in young overweight and obese Chinese adults

**DOI:** 10.3389/fnut.2025.1599603

**Published:** 2025-09-04

**Authors:** Huihe Chen, Shuai Peng, Runa A, Minghui Chen, Lixiu Yuan, Manyun Long

**Affiliations:** ^1^Department of Rehabilitation Medicine, The First Affiliated Hospital of Guangxi Medical University, Nanning, China; ^2^Department of Radiology, The First Affiliated Hospital of Guangxi Medical University, Nanning, China; ^3^Department of Cardiology, The First Affiliated Hospital of Guangxi Medical University, Nanning, China

**Keywords:** overweight, metabolic syndrome, ROC, mediation effects, young adults

## Abstract

**Background:**

Evidence on the combined diagnostic and mediating effects of visceral adiposity index (VAI), triglyceride-glucose index (TyGi), and platelet-to-HDL ratio (PHR) in young overweight and obese adults with metabolic syndrome (MetS) is limited.

**Methods:**

Overweight or obese patient from the Integrated Diagnosis and Treatment Center for Obesity were enrolled. Multivariable logistic regression was used to assess associations between the three markers and MetS severity. Receiver operating characteristic (ROC) analysis evaluated their diagnostic value, and mediation analysis explored their interrelationships.

**Results:**

Among 331 young adults (median age: 31 years; 60% female), the MetS prevalence was 30.2%. Levels of VAI, TyGi, and PHR were significantly higher in participants with MetS and were strongly associated with MetS severity. Their areas under the curve (AUCs) (VAI: 0.825, TyGi: 0.807, PHR: 0.683) outperformed that of waist circumference (0.604). While the combined use of all three markers yielded the highest AUC, it did not significantly exceed that of VAI alone. Mediation analysis revealed complex interrelationships: TyGi had the strongest total effect on MetS (43.3%, *p* < 0.05), with substantial mediation by VAI (58.6%) and PHR (10.4%). The effect of VAI (12.2%) was partly mediated by TyGi (30.8%, *p* < 0.05), whereas the influence of PHR (7.5%, *p* < 0.05) was largely mediated by VAI (68.6%) and TyGi (61.3%).

**Conclusion:**

VAI, TyGi, and PHR are valuable diagnostic markers for MetS in young overweight and obese Chinese adults, with VAI showing the strongest predictive performance. Their interplay highlights the need for integrated interventions targeting visceral adiposity, insulin resistance, and inflammation to mitigate MetS progression.

**Clinical trial registration:**

https://www.chictr.org.cn/index.html, identifier ChiCTR2400082205.

## Introduction

Overweight and obesity have become a global public health crisis, affecting approximately 20% of the population worldwide (1, 2), with an increasing trend among young adults. The high prevalence contributes to the growing burden of metabolic syndrome (MetS), a common comorbidity associated with overweight or obesity (3). Evidence indicates that up to 65% of overweight or obese individuals develop MetS, which increases their risk of cardiometabolic diseases by as much as sixfold (4, 5). Additionally, the annual medical costs of MetS-related conditions continue to rise (6). Therefore, early identification of overweight or obese individuals at high risk for MetS is essential to enable timely management, especially in younger adults who are at a pivotal stage for early intervention.

Visceral fat (7), insulin resistance (IR) (8), and inflammation (9) are recognized as key pathophysiological mechanisms underlying MetS, and several biomarkers associated with these processes have demonstrated significant utility in MetS assessment. Among markers of visceral adiposity, waist circumference (WC) is widely accepted as a diagnostic criterion for MetS in clinical guideline (10). However, the visceral adiposity index (VAI) (11), which integrates both anthropometric and metabolic parameters, has shown a stronger association with MetS than WC alone (12). Similarly, the triglyceride-glucose index (TyGi), a surrogate marker of IR, has been validated as an effective tool for identifying MetS, particularly in middle-aged and older adults (13–15). Recently, increasing evidence also supports the role of inflammation in MetS pathogenesis (16). In addition to traditional inflammatory markers like C-reactive protein (17), novel indices, such as the neutrophil/lymphocyte ratio (NLR) (18) and the neutrophil/high-density lipoprotein ratio (NHR) (19), have been linked to MetS and its individual components. Notably, a study focusing on individuals with newly diagnosed MetS—excluding additional sources of inflammation such as smoking, diabetes, and clinical atherosclerotic cardiovascular disease (CVD)—demonstrated that the platelet/high-density lipoprotein ratio (PHR) is comparable to NLR and NHR in predicting the occurrence of MetS and exhibits superior performance in assessing MetS severity (20, 21).

Despite the robust evidence supporting the independent associations between these indicators and MetS, their discriminative ability varies considerably across studies. For example, the areas under the curve (AUCs) for VAI, TyGi, and PHR in predicting MetS range from 0.6 to 0.8 in various studies (22, 23). To date, no studies have yet explored the synergetic value of these three indicators in combination. More importantly, most studies have primarily focused on middle-aged and older adults (24, 25), thereby overlooking the younger population, which is increasingly vulnerable to MetS and may benefit most from early interventions. Furthermore, most research has concentrated on the occurrence of MetS, with far fewer addressing its severity. This knowledge gap presents a significant barrier to the clinical implementation of MetS diagnosis, as health-care professionals may fail to recognize the importance of early intervention, particularly in younger adults, to prevent the development of cardiometabolic diseases.

In this context, our study aimed to address this gap by utilizing data from young overweight and obese adults at a Class A tertiary hospital. Our objectives were twofold: (1) to explore the associations between visceral fat, IR, and inflammatory markers with the presence and severity of MetS in this population; (2) to investigate the individual and combined effects of these markers in distinguishing the occurrence of MetS; and (3) to evaluate the mediation effects between the three markers and MetS. By focusing on younger adults, this research aims to provide valuable insights for early detection and intervention, ultimately improving long-term cardiometabolic outcomes.

## Methods

### Study design and participants

**Figure 1:**
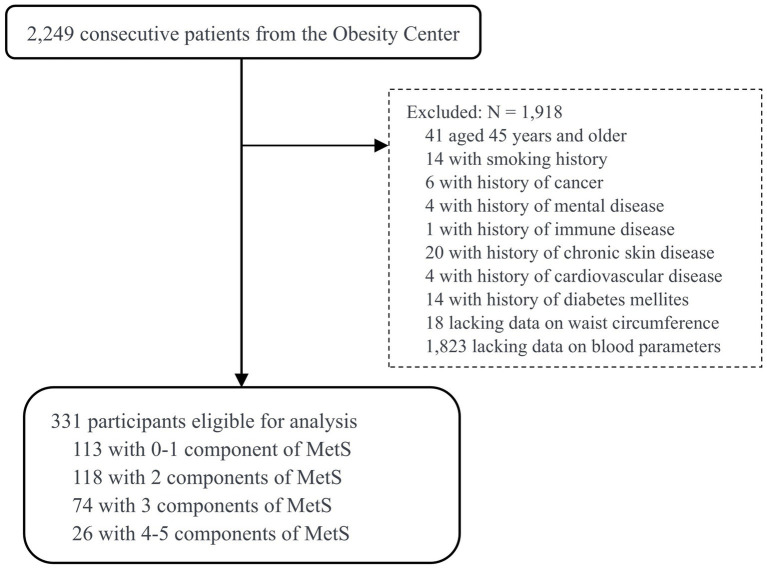
Flowchart of the study design and participant selection process. MetS, metabolic syndrome.

From July 2022 to December 2023, patients with overweight or obesity from the Integrated Diagnosis and Treatment Center for Obesity (short for Obesity Center) at The First Affiliated Hospital of Guangxi Medical University, Guangxi, China, were included in this cross-sectional study. Inclusion criteria were: age between18 and 44 years, and completion of both anthropometric and biochemical assessments. Exclusion criteria included: pregnancy; presence of severe cardiac, cerebrovascular, chronic liver, chronic kidney, or rheumatic immune system disease; and long-term use of corticosteroids or diuretics. Of the 2,249 consecutive patients, 1,823 were excluded due to lacking information on blood parameters, 41 due to being 45 years and older, 14 due to a history of smoking, and 8 due to a history of CVD or diabetes mellitus (DM). Furthermore, 18 participants without measurements of WC were excluded. The BMI values for the remaining participants were no less than 25 kg/m^2^. After applying these criteria, the final study sample included 331 overweight and obese young adults aged 18 to 44 years. Figure 1 illustrates the sample selection process in this study.

### Definition of metabolic syndrome and severity

The diagnostic criteria for MetS were defined according to the guidelines released by Chinese Diabetes Society (26). A diagnosis of MetS can be made when three or more of the following five components were present: (1) abdominal obesity: WC ≥ 85 cm for women and ≥ 90 cm for men; (2) hyperglycemia: fasting plasma glucose (FPG) ≥ 6.1 mmol/L and/or the 2 h plasma glucose ≥7.8 mmol/L and/or with history of DM and using antidiabetic medications; (3) elevated blood pressure: systolic blood pressure (SBP) ≥ 130 mmHg and/or diastolic blood pressure (DBP) ≥ 85 mmHg, and/or with history of hypertension and using antihypertensive therapy; (4) fasting triglycerides (TG) ≥ 1.7 mmol/L; (5) fasting high-density lipoprotein cholesterol (HDL-C) < 1.04 mmol/L. Following prior studies to quantify metabolic burden and predict cardiometabolic risk, MetS severity groups were defined according to the total score of the five abnormalities: non-MetS (as reference and scored 0–1), pre-MetS (scored 2), mild MetS (scored 3), and severe MetS (scored 4–5) (18, 27).

### Clinical assessment

Clinical data were collected after patients arrived at the Obesity Center. Detailed general and medical information was recorded, including age (years), sex (male/female), smoking history (yes/no), regular physical activity (yes/no), and previous physician diagnosis of hypertension, DM, dyslipidemia, or CVD. New diagnoses of hypertension, DM, and dyslipidemia were determined according to the International Classification of Diseases, 10th revision. Hypertension was diagnosed if SBP ≥ 140 mmHg and/ or DBP ≥ 90 mmHg, or if the patient was using antihypertension medication, or had a prior physician diagnosis of hypertension. DM was diagnosed if FPG ≥ 7.0 mmol/L, or if the participant self-reported a diagnosis of diabetes, or was using antidiabetic medication. Dyslipidemia was diagnosed if TG ≥ 1.7 mmol/L, or total cholesterol (TCHO) ≥ 5.2 mmol/L, or HDL-C < 1.04 mmol/L. Long-term prescribed medications were recorded.

Anthropometry was measured during physical examination. Height (cm) and weight (kg) were assessed with patients wearing light cloth and without shoes. WC (cm) was measured using non-elastic tape at the umbilical level after normal expiration. Resting blood pressure of patients was determined using an aneroid sphygmomanometer. Biochemical parameters were assessed using blood sample after overnight fasting. FPG, TG, TCHO, HDL-C, and low-density lipoprotein cholesterol (LDL-C) were determined by standard enzymatic techniques in the hospital laboratory.

Visceral fat, IR, and inflammation were evaluated using VAI, TyGi, and PHR by following equations: (1) VAI: male = [WC (cm)/ (39.68 + 1.88BMI (kg/m^2^))] [TG (mmol/L)/1.03][1.31/HDL (mmol/L)], female = [WC (cm)/(36.58 + 1.89BMI (kg/m^2^))][TG (mmol/L)/0.81][1.52/HDL (mmol/L)]; (2) TyGi = Ln (TG(mg/dL) × FBG(mg/dL)/2); (3) PHR = PLT (1,000 cells/μL) / HDL-C (mmol/L).

### Statistical analysis

Continuous variables were presented as mean (SD) if normally distributed and as median (Q1, Q3) if skewed distributed. Categorical variables were presented as numbers and percentages. Chi-square tests for categorical variables and *t*-tests for continuous variables were used to assess group differences. To ensure comparability, continuous indicators of visceral fat, IR, and inflammation were standardized prior to logistic regression analysis. Multicollinearity was evaluated using the Variance Inflation Factors (VIFs) and no evidence of collinearity was found (VIF < 5, Table S1). Three models were built to investigate the association of visceral fat, IR, and inflammation indicators with MetS. Model 0 was the crude model and only included the target indicator. Model 1 was adjusted for age, PA, SBP, LDL-C, TCHO, dyslipidemia, and hypertension. Model 2 was further adjusted for WC. The odds ratios (ORs) and 95% confidence interval (CIs) were calculated for each model. Receiver operating characteristics (ROC) analysis was employed to calculate the areas under the curve (AUCs) for the occurrence of MetS in the overall study population. Additionally, stratified analyses were performed by gender to assess potential sex-specific differences in predictive performance. Difference between AUCs was examined using De-long test. As VAI, TyGi, and PHR were all significantly associated with MetS, a two-way mediation effect model was used to investigate the mediating roles of each indicator in the pathway to MetS. All analyses were performed using R (version 2023.09.1). A two-sided *p* < 0.05 was considered statistically significant.

## Results

### General characteristics

A total of 331 young adults were analyzed in this cross-sectional study, with a median age of 31 years (IQR: 25.0–36.0) and 60% being female (Table 1). The prevalence of MetS was 30.2%. Compared to the non-MetS group, participants in the MetS group were significantly older (34.0 vs. 31.0 years, *p* = 0.001) and more likely to be male (48.5% vs. 36.3%, *p* = 0.049). The median BMI was significantly higher in the MetS group (34.2 vs. 32.5 kg/m^2^, *p* = 0.008), as was the median WC (109.0 vs. 102.0 cm, *p* = 0.002). Regular exercise was reported by 13.1% of participants, with no significant difference between groups (*p* = 0.093).

**Table 1:** General characteristics of study sample.

Variables	Overall *n* = 331	no-MetS *n* = 231	MetS *n* = 100	*p*
Age, years [median (IQR)]	31.0 (25.0, 36.0)	31.0 (24.0, 36.0)	34.0 (29.0, 37.0)	0.001
Sex, female [*n* (%)]	201 (60.0)	149 (63.7)	52 (51.5)	0.049
BMI, kg/m^2^ [median (IQR)]	33.0 (29.8, 36.3)	32.5 (29.3, 35.8)	34.2 (30.9, 37.6)	0.008
WC, cm [median (IQR)]	104.0 (96.0, 114.0)	102.0 (94.2, 112.9)	109.0 (99.0, 118.5)	0.002
Physical activity [*n* (%)]	44 (13.1)	36 (15.4)	8 (7.9)	0.093
SBP, mmHg [median (IQR)]	130.0 (120.0, 142.0)	128.0 (118.0, 139.0)	133.0 (125.0, 145.0)	0.001
PLT, 1000 cells/Μl [mean (SD)]	303.98 (72.39)	306.74 (74.40)	297.60 (67.43)	0.29
FBG, mmol/L [median (IQR)]	4.7 (4.3, 5.2)	4.6 (4.2, 5.1)	5.0 (4.6, 5.6)	<0.001
TG, mmol/L [median (IQR)]	1.6 (1.2, 2.3)	1.4 (1.1, 1.8)	2.4 (1.8, 3.2)	<0.001
TCHO, mmol/L [median (IQR)]	5.1 (4.5, 5.8)	5.1 (4.5, 5.7)	5.0 (4.4, 5.8)	0.833
LDL-C, mmol/L [median (IQR)]	3.1 (2.7, 3.7)	3.1 (2.7, 3.7)	3.2 (2.7, 4.0)	0.501
HDL-C, mmol/L [median (IQR)]	1.2 (1.1, 1.4)	1.3 (1.1, 1.4)	1.0 (0.9, 1.2)	<0.001
Hypertension, yes [*n* (%)]	33 (9.9)	14 (6.0)	19 (18.8)	0.001
Dyslipidemia, yes [*n* (%)]	60 (17.9)	35 (15.0)	25 (24.8)	0.047
VAI [median (IQR)]	2.3 (1.5, 3.4)	1.9 (1.3, 2.6)	3.6 (2.6, 5.3)	<0.001
TyGi [median (IQR)]	8.7 (8.3, 9.1)	8.5 (8.3, 8.9)	9.2 (8.8, 9.5)	<0.001
PHR [median (IQR)]	6.4 (5.4, 7.9)	6.2 (5.1, 7.1)	7.4 (6.2, 8.6)	<0.001

IQR, interquartile range; MetS, metabolic syndrome; BMI, Body Mass Index; WC, waist circumference; SBP, systolic blood pressure; PLT: platelet count; FBG: fasting blood glucose; TG: triglycerides; TCHO: total cholesterol; LDL-C: low-density lipoprotein cholesterol; HDL-C: high-density lipoprotein cholesterol; VAI, visceral adiposity index; TyGi: triglyceride-glucose index; PHR: platelet to high-density lipoprotein ratio.

Participants in the MetS group had higher systolic blood pressure and poorer glucose and lipid profile. The prevalence of hypertension (18.8% vs. 6.0%, *p* = 0.001) and dyslipidemia (24.8% vs. 15.0%, *p* = 0.047) was significantly higher in the MetS group. Additionally, the VAI, TyGi, and PHR were significantly elevated in the MetS group (*p* < 0.001 for all). No significant differences were observed between the groups in TCHO, LDL-C, or PLT.

### Association of VAI, TyGi, and PHR with MetS severity

Logistic regression analysis showed that the standardized VAI, TyGi, and PHR were significantly associated with different MetS severity groups in all models (Table 2 and Table S2). In general, the ORs in multivariable Model 1 were greater than those in the crude Model 0, and the association remained significant after further adjustment for WC in Model 2. Compared with the non-MetS group, the ORs for each standardized indicator gradually increased across the pre-MetS, mild MetS, and severe MetS groups. In the fully adjusted models, the OR (95% CI) for standardized VAI were 17.4 (7.2–42.1) for pre-MetS, 70.2 (15.9–309.3) for mild MetS, and 326.7 (47.9–2229.9) for severe MetS, respectively. The OR (95% CI) for standardized TyGi were 10.2 (5.1–20.4), 10.2 (5.1–20.4), and 35.7 (12.2–104.7). The OR (95% CI) for standardized PHR were 2.1 (1.5–3.1), 2.5 (1.6–4.1), and 3.6 (2.2–5.8). Overall, the results indicate that VAI, TyGi, and PHR were strong and independently associated with MetS severity, with VAI showing the highest magnitude of association across all models.

**Table 2:** Logistic regression analysis for MetS and its severity.

Variables	pre-MetS^A^			Mild MetS^B^			Severe MetS^C^		
	Model 0	Model 1	Model 2	Model 0	Model 1	Model 2	Model 0	Model 1	Model 2
	OR (95%CI)	OR (95%CI)	OR (95%CI)	OR (95%CI)	OR (95%CI)	OR (95%CI)	OR (95%CI)	OR (95%CI)	OR (95%CI)
VAI^D^	7.6 (4.1–14.3)*	18.2 (7.5–43.9)*	17.4 (7.2–42.1)*	28.4 (10.4–77.4)*	71.8 (16.5–312.7)*	70.2 (15.9–309.3)*	107.7 (28.9–401.7)*	331.0 (49.3–2224.5)*	326.7 (47.9–2229.9)*
TyGi^D^	7.8 (4.5–13.8)*	9.9 (5.0–19.3)*	10.2 (5.1–20.4)*	11.9 (5.9–24.6)*	20.6 (7.8–53.9)*	19.7 (7.5–51.8)*	20.9 (9.3–47.0)	37.3 (12.7–109.1)*	35.7 (12.2–104.7)*
PHR^D^	1.2 (0.9–1.6)	2.1 (1.5–3.0)*	2.1 (1.5–3.1)*	1.8 (1.3–2.4)*	2.7 (1.7–4.3)*	2.5 (1.6–4.1)*	2.3 (1.6–3.2)	3.7 (2.3–6.0)*	3.6 (2.2–5.8)*

Reference group: participants with at most one MetS components. A: defined as having two out of the five MetS components. B: defined as having three out of the five MetS components. C: defined as having no less than four MetS components. D: variables were under standardized. Model 0: only including target indicator. Model 1: adjusted for age, PA, SBP, LDL-C, TCHO, dyslipidemia, and hypertension. Model 2: further adjusted for WC. OR: odds ratio; CI: confidence interval; MetS: metabolic syndrome; VAI: visceral adiposity index; TyGi: triglyceride-glucose index; PHR: platelet to high-density lipoprotein ratio. *: *p* < 0.001.

### ROC analysis and cut-off values

**Figure 2:**
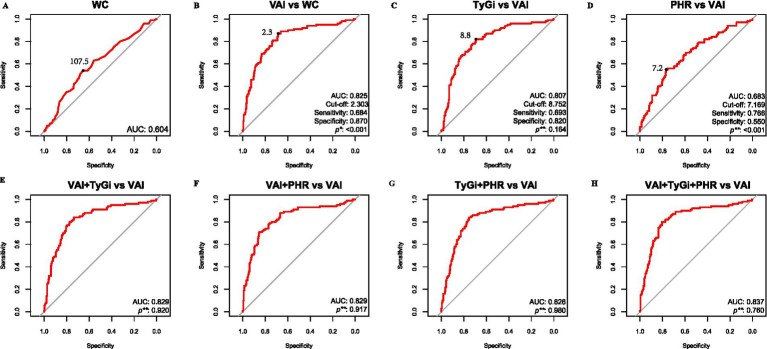
ROC analysis for MetS. **(A)** ROC curve of WC. **(B)** ROC curve of VAI. **(C)** ROC curve of TyGi. **(D)** ROC curve of PHR. **(E)** ROC curve of the combination of VAI and TyGi. **(F)** ROC curve of the combination of VAI and PHR. **(G)** ROC curve of the combination of TyGi and PHR. **(H)** ROC curve of the combination of VAI, TyGi, and PHR. *: De-long test between target AUC marker and WC. **: De-long test of AUC between target marker and VAI. The black dot represents the cut-off value. ROC: Receiver operating characteristic; AUC: area under the curve. WC: waist circumference; VAI: visceral adiposity index; TyGi: triglyceride-glucose index; PHR: platelet-to-HDL ratio.

The discriminative ability of WC, VAI, TyGi, and PHR for MetS was evaluated using ROC analysis (Figure 2). Among the individual results, AUCs of VAI, TyGi, and PHR were 0.825, 0.807, and 0.683, respectively, all significantly greater than 0.604 for WC. The cut-off values were 2.3 for VAI, 8.8 for TyGi, and 7.2 for PHR. All combined models yielded AUCs higher than that of VAI, with the combination of the three indices producing the highest AUC. However, the difference between the combined AUC and that of VAI alone was not statistically significant. Gender-stratified ROC analyses showed that VAI and TyGi had higher AUCs for diagnosing MetS in females than in males, whereas PHR showed a slightly higher AUC in males. Notably, the VAI threshold was higher among females, whereas the thresholds of TyGi and PHR were higher among males (Figure S1 and Figure S2).

### Mediation analysis

**Figure 3:**
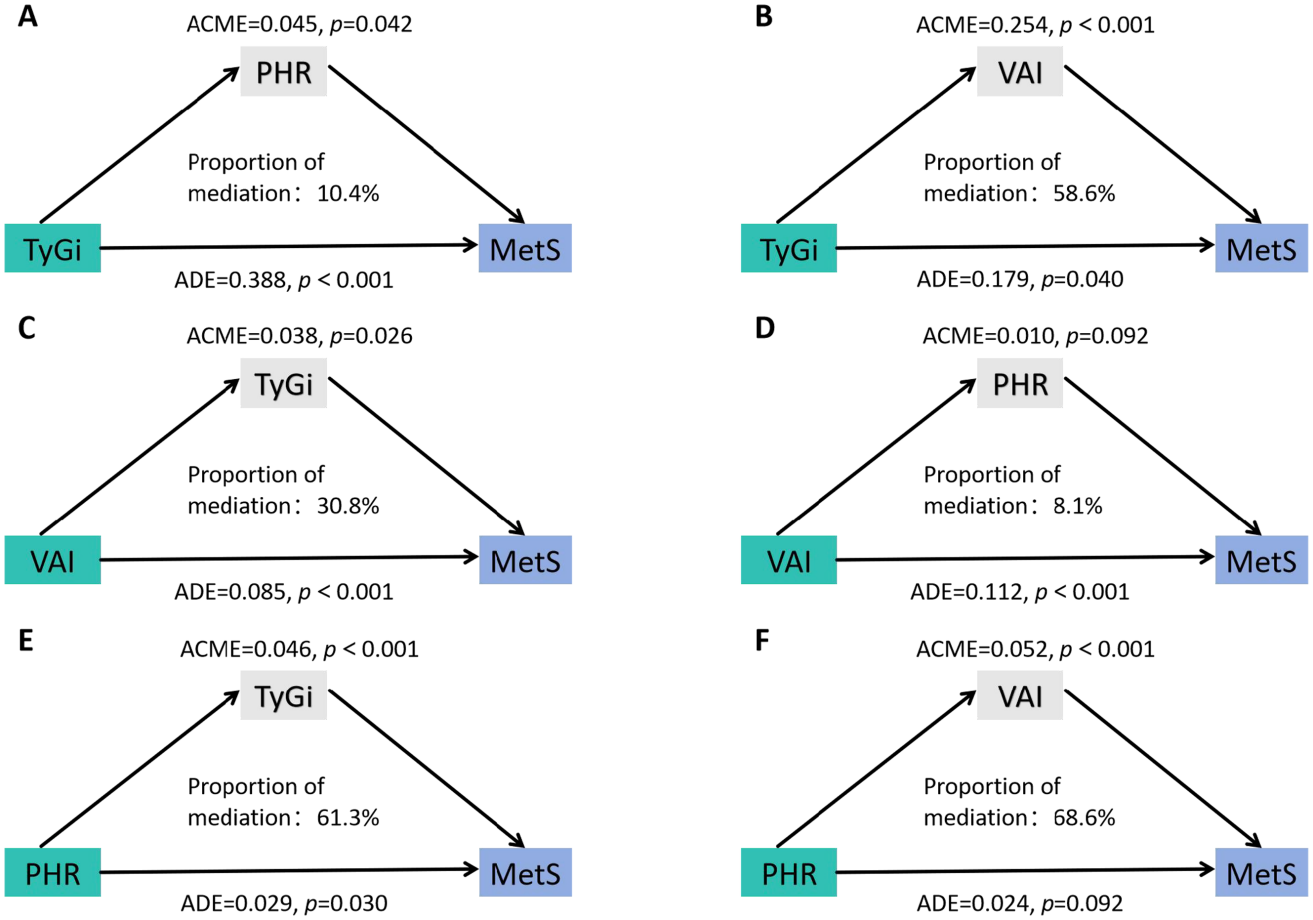
Mediation analysis of the association between target marker and MetS. **(A)** Mediation analysis of the effect of TyGi on MetS with PHR as the mediator. **(B)** Mediation analysis of the effect of TyGi on MetS with VAI as the mediator. **(C)** Mediation analysis of the effect of VAI on MetS with TyGi as the mediator. **(D)** Mediation analysis of the effect of VAI on MetS with PHR as the mediator. **(E)** Mediation analysis of the effect of PHR on MetS with TyGi as the mediator. **(F)** Mediation analysis of the effect of PHR on MetS with VAI as the mediator. VAI, visceral adiposity index; TyGi, triglyceride-glucose index; PHR, platelet-to-HDL ratio; MetS, metabolic syndrome. ACME, average causal mediation effect; ADE, average direct effect.

The bidirectional mediating role of VAI, TyGi, and PHR on their individual relationships with MetS was examined. As shown in Figure 3, the total effect of TyGi on MetS was 43.3% (*p* < 0.05), with 58.6% (*p* < 0.05) mediated by VAI and 10.4% (*p* < 0.05) mediated by PHR. The total effect of VAI on MetS was 12.2%, with 30.8% (*p* < 0.05) mediated by TyGi and 8.1% (*p* > 0.05) mediated by PHR. However, the mediating role of PHR was not statistically significant, suggesting a negligible indirect effect through PHR in the association between VAI and MetS. The total effect of PHR on MetS was 7.5% (*p* < 0.05), with 68.6% (*p* < 0.05) mediated by VAI and 61.3% (*p* < 0.05) mediated by TyGi.

## Discussion

This cross-sectional study assessed the individual and combined utility of VAI, TyGi, and PHR in identifying MetS in young adults. All three indices were independently associated with MetS occurrence and severity. The identified MetS occurrence thresholds were 2.3 for VAI, 8.8 for TyGi, and 7.2 for PHR. VAI exhibited diagnostic performance comparable to the combined use of all three indices, highlighting its robustness as a standalone marker. Mediation analysis revealed complex interrelationships among these indices, showing both direct and indirect effects on MetS. These findings suggest the potential of these three indices in monitoring MetS development, identifying individuals with elevated indices early may enable timely, targeted interventions to prevent disease progression.

Our study demonstrated the intricate interplay among visceral fat, IR, and inflammation in the development of MetS, emphasizing the need for multifaceted intervention strategies. These three factors are tightly interconnected, with IR driving metabolic dysfunction, visceral adiposity exacerbating IR, and inflammation further compounding MetS progression. Mediation analysis revealed that TyGi exerted the strongest total effect on MetS, with a significant portion mediated through VAI, suggesting that targeting IR could influence MetS both directly and indirectly by modulating visceral fat accumulation. In addition to TyGi’s mediation role, VAI and PHR also exhibited complex interdependence, further reinforcing the interconnected nature of these pathways. These interrelationships are not only theoretical but are reflected in real-world treatment strategies. Many anti-diabetic medications, such as metformin (28) and dipeptidyl peptidase-4 inhibitors (29), have demonstrated anti-inflammatory properties by reducing circulating inflammatory proteins and suppressing inflammasomes. Additionally, reducing visceral adipose tissue has been shown to lower systemic inflammation (30), indicating the cumulative effect on metabolic dysfunction. The interplay could be explained by the role of adipose tissue as an active endocrine organ that secretes inflammatory mediators, thereby perpetuating metabolic disturbances (31, 32). These findings highlight the need for an integrated therapeutic approach that concurrently targets IR, visceral fat, and inflammation to effectively mitigate MetS progression.

Although PHR had the weakest direct association with MetS, its significant mediating role suggests that inflammation-targeted interventions may be particularly beneficial for individuals who remain at high metabolic risk despite addressing IR and visceral fat. This aligns with emerging evidence supporting the use of anti-inflammatory agents to improve metabolic outcomes in high-risk populations. Preclinical trials have demonstrated the potential of anti-inflammatory therapies in enhancing glycemic control and reducing IR in obese young adults (33, 34). These findings underscore the importance of a multi-targeted therapeutic approach that not only addresses IR and visceral fat but also considers anti-inflammation as a potential treatment in MetS management.

To our knowledge, this is the first study to evaluate the combined diagnostic utility of three key markers—VAI, TyGi, and PHR—in identifying MetS. The results demonstrated a synergistic effect among these markers in MetS identification, with the combined model achieving the highest AUC compared to any two-marker combination or individual marker. This finding is biologically plausible, as the pathophysiology of MetS is closely associated with insulin IR (35), visceral fat accumulation (36), and low-grade inflammation (37), which are, respectively, reflected by TyGi, VAI, and PHR. Notably, although the three-marker combination showed superior diagnostic performance, the AUC difference between VAI alone and the combined model was not statistically significant. This suggests that VAI alone may be sufficient for identifying MetS in young overweight or obese adults. This observation is consistent with previous studies on middle-aged and older adults (12, 38–40) as well as adolescents (41). Furthermore, in multivariate analysis, the ORs for all three markers increased as the number of MetS components rose, reinforcing their role in MetS severity stratification. VAI had the highest ORs for different MetS severity, even after adjusting for WC. These findings revealed VAI’s potential as a superior tool for assessing MetS severity compared to TyGi and PHR in young adults, a population underrepresented in previous research. Our results align with previous studies, reinforcing VAI’s robust value for MetS screening and monitoring. Moreover, considering established sex-related differences, the observed variation in diagnostic performance and thresholds underscores the need to explore gender-specific thresholds in future research.

Notably, the variability in diagnostic thresholds across studies should be acknowledged. For instance, a US-based study on middle-aged and older adults proposed a threshold of 3.6 (12), whereas our study reported a lower threshold (2.3) for young adults, with even lower values (1.7) in pediatric populations (18). These discrepancies highlight the need for population-specific threshold determinations, suggesting that although VAI has consistent diagnostic utility, its optimal cutoff values vary across demographic groups. The lower VAI threshold in young adults compared to middle-aged and older populations likely reflects differences in adipose tissue distribution, metabolic reserve, and insulin sensitivity. These findings highlight the importance of age-specific reference values to optimize MetS screening and intervention strategies.

The rising prevalence of obesity highlights the urgent need for large-scale and early screening among the young population. Given the simplicity and cost-effectiveness of calculating VAI and TyGi—particularly compared to more complex measures like HOMA-IR—these indices hold significant practical value. Their ease of use makes them particularly advantageous in resource-limited settings, such as primary healthcare facilities and rural areas where advanced diagnostic tools may be unavailable. Notably, VAI can serve as an effective screening and monitoring marker for identifying MetS and assessing its severity, providing a practical approach for early detection and disease management. By incorporating these indices into routine metabolic assessments, clinicians can improve early identification of at-risk individuals, enabling timely lifestyle or pharmacological interventions to prevent MetS progression.

This study has several strengths. First, we applied strict inclusion criteria to ensure the validity of our findings. To better assess the role of inflammation in MetS, we excluded participants who smoked or had acute infections, minimizing potential confounding effects. Second, we evaluated the mutual mediation effects of the target variables, providing deeper insights into their interrelationships. Despite these strengths, the study has several limitations. First, due to its cross-sectional design, this study only establishes associations rather than causal relationships. Additionally, as the sample was drawn from a single Obesity Center in China, the findings may not be generalizable to broader populations. Future prospective studies should validate these findings in diverse populations and explore whether integrating VAI, TyGi, and PHR into clinical risk models improves long-term MetS prediction and intervention efficacy. In addition, the relatively small number of participants in some MetS severity categories may have contributed to the wide CIs and elevated ORs observed, potentially influencing the mediation analysis results. Therefore, these results should be interpreted with caution and validated in larger, more diverse cohorts, particularly for stratified analyses. Another limitation is that some data, such as smoking history, were self-reported, which may have introduced information bias. Lastly, this study involved multiple regression analyses and intergroup comparisons, which may increase the false positive rate due to multiple hypothesis testing. Although formal corrections (e.g., Bonferroni or false discovery rate adjustments) were not applied, the analyses were exploratory in nature, aiming to identify preliminary associations for hypothesis generation. Therefore, the findings should be interpreted with caution. Future studies with confirmatory designs and appropriate statistical corrections are warranted to validate these findings.

## Conclusion

This study highlights the diagnostic value of VAI, TyGi, and PHR in MetS assessment in young overweight and obese Chinese adults, with VAI demonstrating the strongest predictive performance. Furthermore, the intricate interplay among these markers underscores the need for integrated intervention strategies targeting visceral adiposity, IR, and inflammation to effectively manage and mitigate MetS progression.

## Data Availability

The original contributions presented in the study are included in the article/Supplementary material, further inquiries can be directed to the corresponding authors.
