# Supplementary material for: Diagnostic value and mediation effects of the visceral adiposity index, triglyceride-glucose index, and platelet-to-HDL ratio in young overweight and obese Chinese adults
Source: Front Nutr. 2025 Sep 4;12:1599603. doi: 10.3389/fnut.2025.1599603 (PMC12443824; doi:10.3389/fnut.2025.1599603)
Supplement: Supplementary file 3 [file Image_2.pdf]

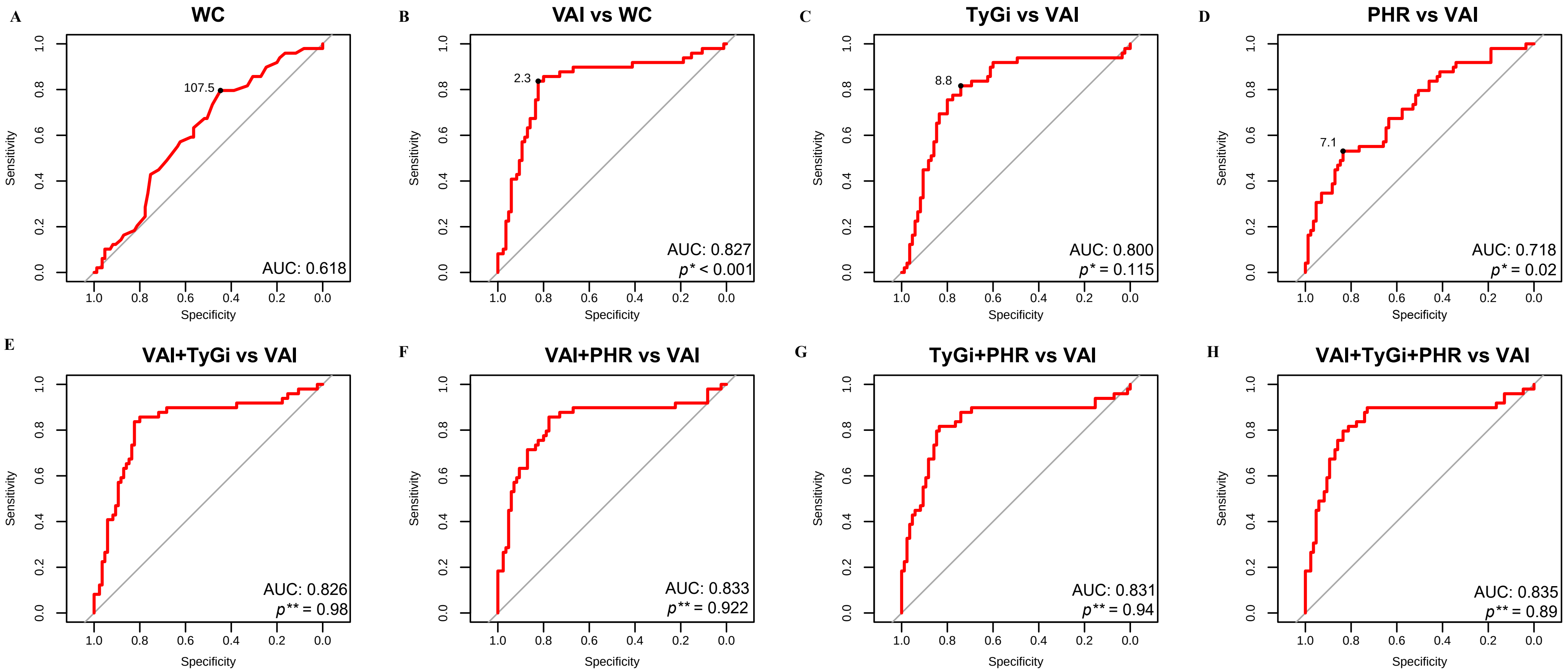

Figure S2: ROC analysis for MetS among males.

(A) ROC curve of WC. (B) ROC curve of VAI. (C) ROC curve of TyGi. (D) ROC curve of PHR. (E) ROC curve of the combination of VAI and TyGi. (F) ROC curve of the combination of VAI and PHR. (G) ROC curve of the combination of TyGi and PHR. (H) ROC curve of the combination of VAI, TyGi, and PHR. *: De-long test between target AUC marker and WC. **: De-long test of AUC between target marker and VAI. The black dot represents the cut-off value. ROC: Receiver operating characteristic; AUC: area under the curve. WC: waist circumference; VAI: visceral adiposity index; TyGi: triglyceride-glucose index; PHR: platelet-to-HDL ratio.
